# Two in one sweep: aluminum tolerance and grain yield in P-limited soils are associated to the same genomic region in West African Sorghum

**DOI:** 10.1186/s12870-014-0206-6

**Published:** 2014-08-12

**Authors:** Willmar L Leiser, Henry Frederick W Rattunde, Eva Weltzien, Ndiaga Cisse, Magagi Abdou, Abdoulaye Diallo, Abocar O Tourè, Jurandir V Magalhaes, Bettina IG Haussmann

**Affiliations:** Institute of Plant Breeding, Seed Science and Population Genetics, University of Hohenheim, Fruwirthstr. 21, 70599 Stuttgart, Germany; International Crops Research Institute for the Semi-Arid Tropics, P 320, Bamako, Mali; Institut Sénégalais de Recherches Agricoles, BP 3320, Thiès, Senegal; Institut National de la Recherche Agronomique, CERRA de Maradi, BP 240, Maradi, Niger; L’Institut d’Economie Rurale, BP 258, Bamako, Mali; Embrapa Maize and Sorghum, Rod. MG 424, Km 65, 35701-970 Sete Lagoas, Minas Gerais Brazil

**Keywords:** Sorghum, Phosphorus, Aluminum, Breeding, Genetics, West Africa

## Abstract

**Background:**

Sorghum (*Sorghum bicolor* L. Moench) productivity is severely impeded by low phosphorus (P) and aluminum (Al) toxic soils in sub-Saharan Africa and especially West Africa (WA). Improving productivity of this staple crop under these harsh conditions is crucial to improve food security and farmer’s incomes in WA.

**Results:**

This is the first study to examine the genetics underlying sorghum adaptation to phosphorus limitation in a wide range of WA growing conditions. A set of 187 diverse sorghum genotypes were grown in 29 –P and + P field experiments from 2006-2012 in three WA countries. Sorghum grain yield performance under –P and + P conditions was highly correlated (r = 0.85***). Significant genotype-by-phosphorus interaction was detected but with small magnitude compared to the genotype variance component. We observed high genetic diversity within our panel, with rapid linkage disequilibrium decay, confirming recent sequence based studies in sorghum. Using genome wide association mapping based on 220 934 SNPs we identified one genomic region on chromosome 3 that was highly associated to grain yield production. A major Al-tolerance gene in sorghum, *SbMATE*, was collocated in this region and *SbMATE* specific SNPs showed very high associations to grain yield production, especially under –P conditions, explaining up to 16% of the genotypic variance.

**Conclusion:**

The results suggest that *SbMATE* has a possible pleiotropic role in providing tolerance to two of the most serious abiotic stresses for sorghum in WA, Al toxicity and P deficiency. The identified SNPs can help accelerate breeding for increased sorghum productivity under unfavorable soil conditions and contribute to assuring food security in WA.

**Electronic supplementary material:**

The online version of this article (doi:10.1186/s12870-014-0206-6) contains supplementary material, which is available to authorized users.

## Background

Sorghum (*Sorghum bicolor* L. *Moench*) is a staple crop of the Savannah Zone of West- and Central Africa, where it is cultivated in low input cropping systems [1]. Limited P availability and aluminum (Al) toxicity in the soil are serious and frequent constraints to sorghum growth and productivity across sub-Saharan Africa, particularly in West Africa (WA) [2–4]. The adaptation of sorghum to these conditions is crucial for food security, and increasingly for farmer’s income. Sorghum breeding specifically under P-limited conditions, carried out in WA since the last decade, was shown to be necessary and feasible to obtain superior genotypes for these conditions [5]. However, the genetic basis underlying grain yield performance under P-limited conditions in sorghum is not yet known. In contrast, breeding for Al-tolerance in sorghum has received much more attention and the underlying genetic mechanism has been identified. A gene in the multidrug and toxic compound extrusion (MATE) family, *SbMATE*, underlies the *Alt*_*SB*_ locus on chromosome 3, which is mainly responsible for Al-tolerance in sorghum via Al-induced root citrate release [6]. Al-toxicity inhibits root growth and function, which leads to severe crop-yield losses [7]. Release of citrate and other organic anions into the rhizosphere prevents root damage by chelating Al^3+^. At the same time, citrate can mobilize P that is bound to soil clays by ligand exchange, dissolution and occupation of sorption sites, thus increasing P availability to the plants [8–10]. Hence, exudation of organic anions can lead to a higher P acquisition rate [11,12]. Recent work showed that over-expression of citrate synthesis and malate transporter genes in different species resulted in improved Al-tolerance and enhanced P uptake under low P conditions [13–15], supporting previous hypothesis that Al-tolerance and P uptake can be regulated by similar mechanisms [6]. However, in soybean and rape, citrate release is predominantly induced by Al and to a much lesser extent by low P conditions [16–19]. Evaluation of sorghum genotypes under P-limited and slightly Al-toxic soils, conditions common in WA, could shed light on the possible pleiotropic role of *SbMATE* in Al tolerance and P-efficiency and provide new possibilities for enhancing grain yield of sorghum under the prevalent low-input conditions in WA.

We provide the first evidence for a genetic and possible molecular link between Al-tolerance and P-efficiency, opening possibilities for the use of molecular breeding tools to facilitate development of cultivars with superior performance for poor WA soil conditions. We conducted an unprecedented evaluation of WA sorghum genotypes, with 187 West African genotypes assessed for grain yield in 15 low P (–P) and 14 P-fertilized (+P) field experiments in Mali, Niger and Senegal from 2006-2012. Although fertilized, some of the + P fields showed also slight P limitations, due to the prevailing soil conditions in WA. The genetic architecture of grain yield under –P and + P conditions was investigated using both genome wide association mapping with 220 934 SNPs, obtained by genotyping-by-sequencing (GBS), and gene specific markers associated with Al-tolerance within *Alt*_*SB*_. Significant SNPs close to (<40 kb) and within *Alt*_*SB*_ were associated to grain yield under P-limited conditions, suggesting that *Alt*_*SB*_ has a pleiotropic role in providing tolerance to two of the most important abiotic constraints to sorghum productivity in WA, Al toxicity and P deficiency.

## Results

### Performance across –P and + P environments

**Figure 1:**
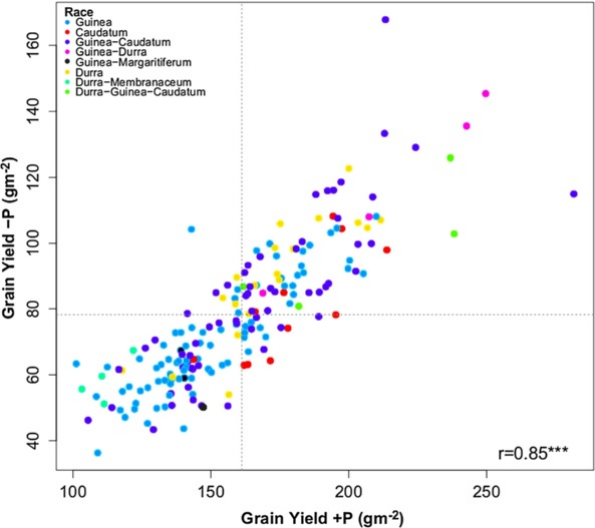
**Scatter plot between grain yield performance (BLUPs) of 187 sorghum genotypes estimated across sites in Mali, Niger and Senegal in 15 –P environments versus 14 + P environments, with genotype dots color-coded for their morphological race-classification.**

Significant grain yield (52%) and plant height (22%) reduction and delayed heading (29%) occurred under –P relative to the + P conditions. The individual –P sites had generally lower repeatabilities for grain yield than the + P sites (Additional file 1: Table S1) but broad sense heritability (h^2^) computed across the 15 –P sites was slightly higher than for the 14 + P sites (Table 1), due to proportionally smaller genotype-by-environment interactions (GxE) among –P environments. Most of the variation for grain yield in –P as well as in + P conditions could be attributed to genotypic effects, with variance component ratios (G:GxE) being greater than one. The genotype-by-P (GxP) interaction variance component estimated across all 29 sites was significant but small. No distinct mega-environments could be delineated in a GGE-biplot analysis (only the 8 common checks were used, see Additional file 2) and the correlation between –P and + P grain yield performance (Figure 1) was high (r = 0.85***), indicating strong correspondence of genotypic performance for grain yield between –P and + P conditions. Therefore best linear unbiased predictions (BLUPs) for grain yield across all environments for each genotype could be used for further analyses. Additionally to the grain yield BLUPs across all environments, we also used grain yield BLUPs estimated only across –P or + P conditions in order to assess genomic regions associated specifically to grain yield under either –P or + P conditions (Table 2). Although genotypes from different sorghum races showed wide variation for grain yield performance in both fertility conditions (Figure 1), the Caudatum accessions showed somewhat more specific adaptation to + P conditions whereas the Durra and Durra inter-racials accessions were generally better adapted to –P conditions (Figure 1, Additional file 3).

**Table 1:** **Variance components (±se) and broad-sense heritability (h** ^**2**^ **) for grain yield of 187 sorghum genotypes analyzed separately within 15 –P and 14 + P environments and combined across P treatments**

**Term**	**–P**	**Combined**	**+P**
**σ** ^**2**^_**G**_	701*** ± 90		1388*** ± 183
**σ** ^**2**^_**GxE**_	423*** ± 31		988*** ± 70
**h** ^**2**^	0.79		0.76
**σ** ^**2**^_**G**_		983*** ± 123	
**σ** ^**2**^_**GxE**_		503*** ± 35	
**σ** ^**2**^_**GxP**_		47*** ± 13	
**σ** ^**2**^_**GxExP**_		101*** ± 17	
**h** ^**2**^		0.81	

G = Genotype, E = Environment (=Location x Year), P = P treatment; *** = significant at <0.001 probability-level.

**Table 2:** **Association results of four SNP markers within the** ***Alt***_***SB***_ **locus with grain yield in + P, –P and –P/+P grain yield ratio across 187 sorghum genotypes**

**Condition**	**Marker**	**p-value**	**R** ^**2**^
**+P**	AltSB_24804	1.56e^−05^	0.12
**+P**	AltSB_6083	0.62	0.01
**+P**	AltSB_8364	0.37	0.00
**+P**	AltSB_8423	0.35	0.00
**-P**	AltSB_24804	1.65e^−07^	0.16
**-P**	AltSB_6083	0.09	0.03
**-P**	AltSB_8364	0.03	0.02
**-P**	AltSB_8423	0.03	0.03
**-P/+P**	AltSB_24804	0.02	0.04
**-P/+P**	AltSB_6083	1.77e^−04^	0.09
**-P/+P**	AltSB_8364	0.001	0.06
**-P/+P**	AltSB_8423	0.001	0.06

### Genetic markers and linkage disequilibrium

**Figure 2:**
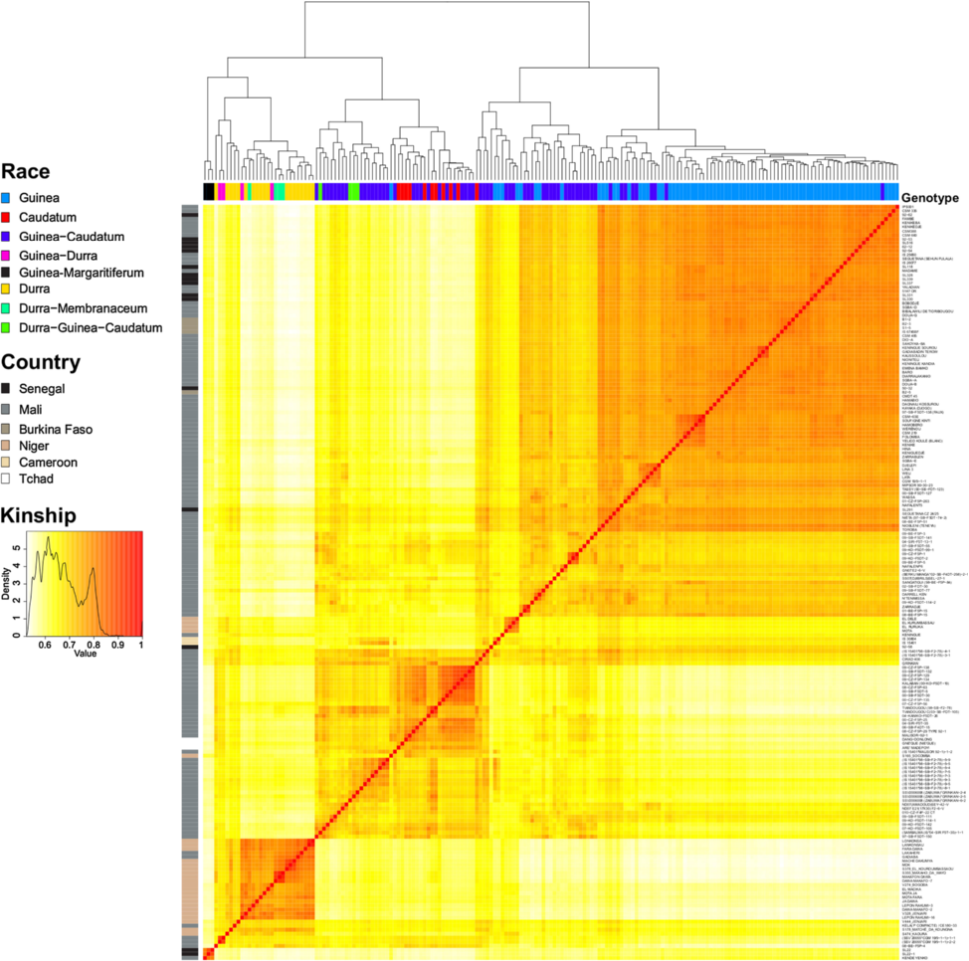
**Heatmap and dendrogram of a kinship matrix estimated using the EMMA algorithm based on ~220 k SNPs among 187 sorghum genotypes from West Africa.** Color codes show the genotype classifications for their morphological race and country of origin. The color histogram (Kinship) shows the distribution of coefficients of coancestry values in the whole kinship matrix.

The diversity panel consisted of 187 sorghum genotypes from six West and Central African countries, consisting of eight racial groups (3 major- and 5 intermediate-racial groups) (Figure 2, Additional file 1: Table S1, Additional file 4) and included breeding lines and landraces, with differing degrees of photoperiod sensitivity and internode lengths. Most of the genotypes are currently used in breeding programs in the region, but there are no clear records for ancestry or precise geographic origin of the majority of these genotypes. All genotypes were genotyped using genotyping-by-sequencing (GBS) [20] yielding 220 934 SNPs after filtering for 5% minor allele frequency (MAF) and imputing missing SNPs, giving an average density of one SNP per 3.1 kbp. Linkage disequilibrium (LD), calculated for each chromosome separately, decayed rapidly to 50% of its initial value across all chromosomes within less than 1 kb (Additional file 5). The high SNP density and the fast LD decay gave a solid basis for genome-wide-association mapping (GWAS).

### Relatedness and population structure

The diversity panel used in this study consisted of five major groups based on kinship and population structure (Figure 2, Additional files 6 and 7). The clustering was primarily associated with differences for morphological race but not for geographic origin. The Guinea and Durra race genotypes formed two very distinct clusters, whereas the Caudatums could be considered intermediate between these two clusters. Generally the inter-racial genotypes were intermediate to their major race clusters, showing different levels of admixture (Additional file 7). A distinct sub-group of Guinea genotypes consisting of the Guinea-margaritiferums clustered closer to the majority of Durra rather than Guinea genotypes, but could only be detected in an admixture analysis using seven, and not the optimum five clusters (Figure 2, Additional file 7).

### GWAS for grain yield

Grain yield BLUPs estimated across 15 –P, 14 + P, combined across all 29 environments and the grain yield ratios (–P/+P) were tested for association to 220 934 SNPs using a mixed model as described in [21]. Several models were compared and the models correcting for kinship (K) and two to three principal components (PCAs) fitted our data best, had the lowest inflation-factor (λ) and therefore the lowest false-discovery rate.

**Figure 3:**
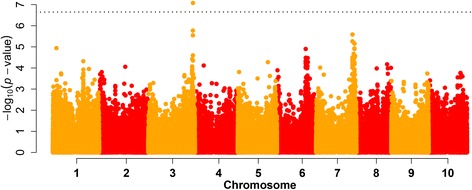
**Manhattan plot for sorghum grain yield BLUPs across 29 environments combined over –P and + P conditions of 187 genotypes genotyped with ~220 k SNPs with** ***P*** **values shown on a log**_**10**_ **scale and Bonferroni threshold at p < 0.05 indicated with dashed line.**

**Figure 4:**
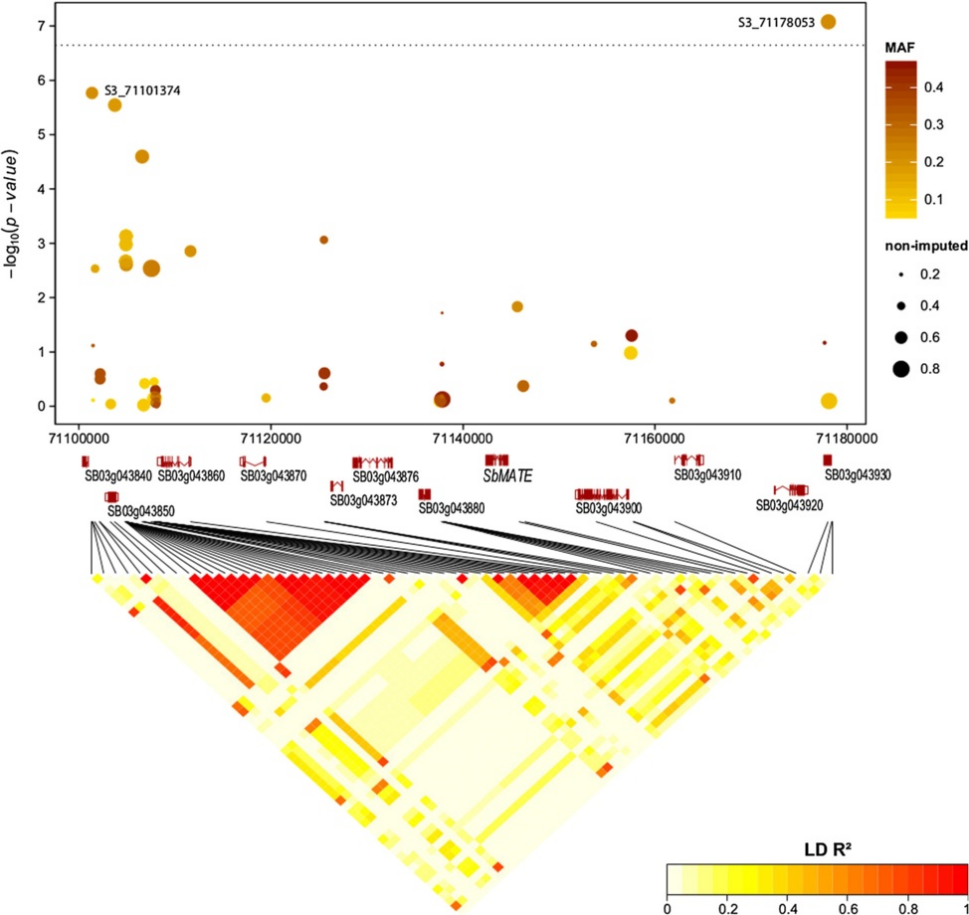
**Associations of SNPs within a 78 kb region on chromosome 3 grain yield BLUPs across 29 environments combined over –P and + P conditions, with annotated genes, LD among SNPs, imputation rate (dot size) of SNPs and minor allele frequency (color range) of SNPs across 187 sorghum genotypes.** *P* values are shown on a log_10_ scale and Bonferroni threshold at p < 0.05 is indicated with dashed line.

We identified one SNP (S3_71178053) for grain yield across –P and + P environments that was significant based on a p < 0.05 Bonferroni threshold (Figure 3) and explained 15% of the genotypic variance. The mean yield of accessions harboring the A allele was 44 g m^−2^ (=440 kg ha^−1^) higher than that of accessions with the G allele, a difference equivalent to a 37% yield increase of the A- over the G-allele genotypes (Additional file 8). Three other SNPs with significant association probabilities with grain yield (–log_10_(p) > 5) were detected at a physical distance less than 78 kb and were in tight LD (R^2^ > 0.65) with S3_71178053 (Figure 4). All other GBS derived SNPs within this region had low –log_10_(p)-values, which was not caused by lower MAF or higher imputation rates (Figure 4) and were not in strong LD with S3_71178053.

Regions on chromosome 6 and 7 also showed strong associations to grain yield (Figure 3). Nine SNPs in gene Sb07g023120 and one SNP (S7_57976035) in gene Sb07g023130 of chromosome 7 were highly associated (–log_10_(p) > 4.5). None of the highly associated SNPs on chromosome 6 were located within coding regions. Grain yield BLUPs estimated only within –P or + P environments tended to show similar associations, but these single P-level associations had less power in detecting significant SNPs (Additional file 9). S3_71178053 was the most significant SNP for grain yield under both –P and + P conditions, whereas we observed differences across the other chromosomes depending on the P-level. SNPs on chromosome 6 played a more important role under –P conditions, whereas chromosome 10 exhibited a region highly associated to grain yield under + P conditions (Additional file 9). Examination of –P/+P grain yield ratios as an indicator for specific adaptation to –P conditions revealed one single SNP on chromosome 5, although not located in a coding region, and a second SNP on chromosome 1, S1_54947742 (–log_10_(p) > 4.5), which caused a missense variant in gene Sb01g032090, leading to an amino acid change from methionine to isoleucine (Additional file 10: Table S2).

### Candidate gene association

**Figure 5:**
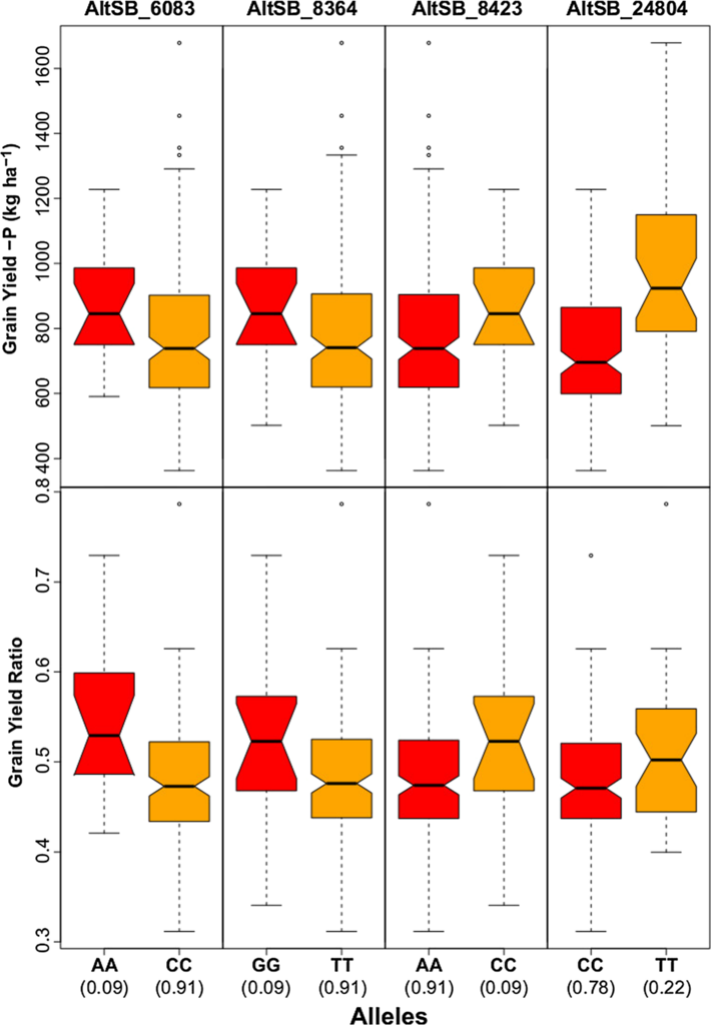
**Fitted values of grain yield of the two homozygous genotypes at each of the significant SNP positions in** ***Alt***_***SB***_ **.** Allele frequencies are given between brackets.

Twelve coding regions, five with putative gene functions and seven uncharacterized proteins, occur within the 78 kb spanning region on chromosome 3 between the SNPs S3_71101374 and S3_71178053 (Figure 4). Only five out of 62 SNPs within this region were located within coding regions and only two out of these five SNPs were highly associated to grain yield performance. The Al-tolerance gene *SbMATE* (Sb03g043890) is also located in the same region. As no SNPs from the GBS dataset were located in *SbMATE*, we used *Alt*_*SB*_ specific SNPs within *SbMATE* and in close vicinity to *SbMATE* [22] to test for association to grain yield under –P and + P conditions and to the –P/+P grain yield ratio. Four of the nine *Alt*_*SB*_ specific SNPs showed promising associations; three of which showed –P specific associations and the fourth, AltSB_24804, had the strongest association to grain yield under –P as well as + P conditions (Table 2, Figure 5). AltSB_24804 showed the highest minor allele frequency and explained 12 and 16% of the genotypic variance for grain yield under + P and –P conditions, respectively, resulting in a 37 g m^−2^ and 25 g m^−2^ grain yield increase, respectively. However, this SNP explained only 4% of the genotypic variance for –P/+P grain yield ratio, indicating no –P specificity. The other three SNPs located within or in close proximity to *SbMATE* explained 6-9% of the genotypic variance for grain yield ratio, significantly increased mean grain yield ratio from 0.47 to 0.54 and increased –P grain yield from 77 to 86 g m^−2^. AltSB_24804 was in strong LD (r^2^ > 0.7) with the four SNPs (–log_10_(p) > 5) on chromosome 3, whereas the other *Alt*_*SB*_ specific SNPs showed no LD based on squared correlation of allele frequencies (r^2^ < 0.02). However, this might be due to unmatched allele frequencies, since D’ values (D’ > 0.7) indicated strong LD.

## Discussion

### Field experiments

The large grain yield and plant height reductions and the delay in flowering in the –P compared to the + P conditions indicate important growth differences between the two P treatments, that should permit a genetic study of adaptation to P-limited conditions [5]. Although the –P trials always had significantly lower grain yields than the + P trials, some P limitations in + P trials cannot be discounted, especially for three + P trials due to the high P-retention in these low pH soils and late application of P fertilizer, thus reducing the impact of P fertilizer application on plant development and final grain yield [23]. Despite an average 52% grain yield reduction in –P relative to + P conditions, the sorghum genotypes exhibited a strong correspondence between –P and + P grain yield performance (r = 0.85***). This suggests that WA sorghums have a general good adaptation to P-limited conditions and corresponds with previously reported higher P uptake and use efficiency of sorghum compared to other crops [5,24–29]. The strong correlation between –P and + P conditions and the small GxP variance component suggest that selection under + P conditions could result in gains under –P conditions. Nevertheless, the slightly higher broad sense heritability in –P (Table 1) and the specific adaptation of some genotypes towards –P conditions (Figure 1, Additional file 3) suggests that direct selection in –P conditions is more efficient for targeting P-limited production systems, as was shown by [5].

The phenological and racial diversity of our panel was so wide that the entire set would not normally be cultivated in the same environments. The Durra-race genotypes and very early maturing Caudatum-race genotypes are cultivated in the Sahelian zone whereas the Guinea-race genotypes are grown in the Sudanian zone of WA [30,31]. Our field trials were therefore designed to evaluate accessions in their respective zone of adaptation. The relatively small GxE-interaction and lack of clear mega-environment delineations based on zones (Additional file 1: Table S1, Additional file 2) may be due, in part, to the experimental design in which only eight Guinea, Caudatum and Guinea-Caudatum genotypes with acceptable adaptation to both zones were common across all environments, hence only these common genotypes actually displayed the GxE-interaction pattern.

### Genetic diversity

Genetic diversity among the 187 WA sorghum genotypes was high, with considerable separation of the three races, but no separation due to geographic origin. Studies showing clear geographic differentiation are mostly based on worldwide collections [32–34], whereas studies considering germplasm from within a smaller region find mostly racial differences [35]. A group of Guinea-margaritiferum accessions was clearly separated from the other races, but clustering closer to the Durra- rather than other Guinea-race accessions, confirming the genetic distinctness of this Guinea-sub-race [32,34,36]. Although there were clear racial differentiations, we also observed a high level of genetic admixture (Additional file 7) in our diversity panel, suggesting that considerable gene flow has occurred in the WA region and thus providing a high genetic diversity exploitable in GWAS. Furthermore, the rapid LD decay, in agreement with recent studies based on GBS [34] and re-sequencing data [36] of diverse sorghum genotypes additionally points to the high genetic diversity of our panel and its usefulness for GWAS with a mapping resolution on gene-level.

The –P specific adaptation and general good performance of the Durra genotypes (Figure 1, Additional file 3) makes this germplasm group particularly promising for use in future sorghum breeding efforts. Their genetic distinctness from the Guinea pool suggest, that Durra by Guinea-race crossing could be explored to achieve high heterosis and to obtain broader adaptation [37] if acceptable grain and glume types can be obtained from these inter-racial matings.

### Genomic regions for grain yield under P-limited conditions

This study, the first to our knowledge, identifies regions in the sorghum genome related to grain yield under P-limited soils and a genomic region with a possible pleiotropic effect for Al-tolerance and grain yield productivity under P-limited conditions. Recent work show that over-expression of citrate synthesis and malate transporter genes improve Al-tolerance and P uptake under low P conditions in several species [13–15], supporting the hypothesis that Al-tolerance and P uptake under low P conditions can be regulated by similar processes [6]. However citrate release is predominantly induced by Al and to a much lesser extent by low P conditions [16–19]. Al-saturation values between 10-28% were found in six out of 16 field trials analyzed for Al-saturation (Additional file 11: Table S3), values which are at or near the threshold for limiting sorghum root growth due to Al-toxicity. Thus, although the Al-stress may have been mild, these conditions could have induced *SbMATE* expression that increased citrate release and benefitted P availability and retention of viable roots [12], thus leading to an increased P uptake. Furthermore, Al saturation shows generally a high spatial variation [38] and has been reported to have a major influence on crop growth in WA [39], therefore an impact of Al toxicity on crop growth in the other environments cannot be ruled out. This might have also lead to the observed strong association of grain yield from both P-treatments to the same genomic region.

The strong effect of *SbMATE* specific SNPs and their rather tight linkage to the other significant SNPs within the 78 kb region identified by GWAS suggests that grain yield under P-limited conditions is possibly controlled by *SbMATE* and likely other genes in this region. S3_71178053, the SNP with the strongest association to grain yield caused a missense mutation in gene Sb03g043930, leading to an amino-acid change from threonine to alanine (Additional file 10: Table S2). Since Sb03g043930 is an uncharacterized protein, it is not clear how it might be involved in grain yield production or *SbMATE* regulation. Recent evidence shows that the *SbMATE* expression in sorghum is regulated by additional factors [40] and thus its expression is more complex than previously thought. The linked SNPs identified in our study could be valuable for studying these background factors. The C2H2-type zinc-finger transcription factors (STOP 1 and 2) regulate MATE expression in *A. thaliana* [41,42], and orthologous genes to STOP1 have been found in several other crops [43]. Other C2H2-type zinc finger genes are also known to play a role in P adaptation and are mostly locally regulated (e.g. roots), whereas C3HC4-type zinc-finger genes are systematically regulated [44] and act as transcription factors in early low P adaptation [45] and other abiotic stress adaptation [46]. SNP S3_71101374, which was highly associated to grain yield performance (Figure 4) and in strong linkage to AltSB_24804, is located close to Sb03g043850, which is predicted to be a C3HC4-type zinc-finger gene (Additional file 10: Table S2), hence possibly involved in adaptation to P-limited conditions. The highly associated *AltSB* specific SNP AltSB_24804, located in the 3´ region of *SbMATE*, was shown to be associated to Al-tolerance in another study, although showing rather small effects [22]. The other three SNPs within or closer to *SbMATE*, especially AltSB_6083, which showed specific responses to –P conditions, are known to be highly responsible for Al-tolerance in sorghum [22], pointing to a possible pleiotropic regulation of Al-tolerance and specific –P adaptation. However, we did not evaluate this set of genotypes specifically for Al-tolerance in soil-based or hydroponic systems, but previous studies showed a very strong effect of AltSB_6083 on phenotypic Al-tolerance, that support this hypothesis. Although the frequency of these positive –P specific SNPs was low (9%) in our WA panel, the frequency appears to be much higher in the Guinea-race germplasm, with 95% of the genotypes carrying the positive *SbMATE* alleles being either pure Guinea race or Guinea introgressed genotypes. The low MAF and confounding of population structure might have limited a stronger association of these SNPs to low P grain yield [22]. A similar allele frequency (0.1) of these SNPs and a higher frequency in WA Guinea sorghums was also found in a worldwide sorghum collection [47], hence pointing to this germplasm group as an important source for Al-tolerance in sorghum [22].

The rather high association on chromosome 7 to grain yield under both –P and + P conditions may have been caused by an unidentified foliar disease resistance gene. The SNP with the best association to grain yield was located within an uncharacterized protein Sb07g023120 but was also in strong LD (R^2^ > 0.85) with SNPs in Sb07g023130. Sb07g023130 is similar to NADPH HC toxin reductase, which confers specific resistance reaction to Helminthosporium (*Cochliobolus carbonum*) in maize [48]. Although *Cochliobolus carbonum* is not known as a major sorghum disease in WA, it is known to cause foliar diseases in sorghum [49] and the HC toxin reductase mechanism is also conserved in other grasses and acts as resistance response to *Cochliobolus carbonum* [50]. Although foliar disease scoring was not done in our study to enable further investigation, the effect on chromosome 7 being expressed under both + P and –P conditions would be consistent with some type of biotic resistance.

Specific adaptation to P-limited conditions was assessed by the –P versus + P ratio of grain yield performance. Although no genomic region was found with strong associations to –P/+P grain yield ratio, SNPs with –log_10_(p) > 4.3 may indicate the existence of mechanisms involved in specific –P adaptation other than *SbMATE*. One of these, SNP S5_2179409 on chromosome 5 was located in an intron region of gene Sb05g001996. Sb05g001996 is predicted to be of the rhamnogalacturonan lyase family, hence possibly involved in cell wall degradation by disrupting rhamnogalacturonan [51]. This might imply that Sb05g001996 is involved in specific low P adaptation by cell wall degradation and consequently in P translocation at later developmental stages. Somewhat similarly, S1_54947742 causes a missense variant in Sb01g032090, which is predicted to be a papain family cysteine protease. Papain family cysteine proteases are known to be involved in programmed cell death, either due to senescence and therefore nutrient cycling or as pathogen defense [52,53]. Phosphorous translocation from shoot to grain is known to be of major importance in sorghum, and P harvest index is increased under P-limited conditions [24]. The existence of significant genotypic differences for specific –P grain yield, measured as –P/+P grain yield ratio (unpublished data), but no identification of a genomic region with clear effects for this specific –P/+P grain yield ratio may result from the existence of multiple adaptive mechanisms contributing to this complex trait.

### Implications for applied breeding

Our test sites represent a wide range of soils and climate conditions relevant to sorghum production in WA. It is thus remarkable that we found individual SNPs accounting for up to 16% of the genotypic variation for grain yield. The significant GxP interaction, higher response to direct selection under –P conditions [5] and the low frequency of Al-tolerance in WA sorghum, indicate the need for selection efforts specifically targeting P-limited and high Al-saturation production environments, which are of major importance for WA sorghum producers. Our results suggesting that the same genomic region or even the same gene, *SbMATE*, possibly contribute to adaptation to both conditions focuses attention on this region. Currently we are evaluating further diversity and bi-parental mapping sets for performance under low P conditions to validate these findings. Once validated, it should be possible to incorporate the desired loci for both traits into elite breeding material with minimum linkage drag using marker assisted selection. Furthermore, the gene specific markers within *Alt*_*SB*_ will make allele mining possible in large germplasm collections, hence increase allelic frequency and Al-tolerance of the currently used germplasm. These markers are already available on the Integrated Breeding Platform of the Generation Challenge Program [https://www.integratedbreeding.net/]. All other markers, once validated, will be also accessible on this platform to help sorghum breeders select promising genotypes for two traits in one sweep.

## Conclusions

We provide the first evidence for a genetic and possible molecular link between Al-tolerance, P-efficiency and grain yield potential, opening possibilities for the use of molecular breeding tools to facilitate development of cultivars with superior performance under the predominant poor soil conditions of WA. The significant GxP interaction, higher response to direct selection under –P conditions and the low frequency of Al-tolerance in WA sorghum, indicate the need for selection efforts specifically targeting P-limited and high Al-saturation production environments. Our results point to the possibility that adaptation to both conditions is pleiotropically controlled by the same genomic region or even the same gene, *SbMATE*. The identified SNPs can help accelerate breeding for increased sorghum productivity under the two most important abiotic constraints to sorghum productivity in WA, Al toxicity and P deficiency, and contribute to assuring food security in WA.

## Methods

### Field trials

A total of 187 sorghum genotypes from six West and Central African countries, consisting of researcher-bred and landrace varieties representing eight racial groups (Guinea, Durra, Caudatum and 5 intermediate groups; Additional file 4) and differing degrees of photoperiod sensitivity and stem internode lengths, were grown in paired –P (0 kg P ha^−1^ application) and + P (20-40 kg P ha^−1^ application, Additional file 11: Table S3) experiments in each location-year combination. A total of 29 rain fed field experiments (Additional file 1: Table S1), consisting of 15 –P and 14 + P environments, were successfully conducted in three countries (Mali, Niger, Senegal; Additional file 12) at five locations over seven years (2006-2012). Due to field size constraints (max. 320 plots per experiment) and specific adaptation requirements for the Sahelian and Sudanian zones, three separate series of trials were conducted with subsets of the 187 genotypes (Additional file 1: Table S1). At least eight common genotypes considered to have acceptable adaptation to both the Sudanian and Sahelian zones were included in all experiments to enable a combined analysis across all sites, hence fit BLUPs for each genotype across all environments and estimate genotype-by-environment interaction. The set 1 genotypes, consisting of 36 landrace and 34 research-bred varieties originating from the Sudanian zone, were tested at two locations in the Sudanian zone of Mali from 2006-2010 in the first series of trials comprising eight –P and seven + P environments [5]. Set 2 genotypes, consisting of 54 landraces from the Sahelian zone and 28 early maturing research-bred varieties, were evaluated at four Sahelian zone locations in Mali, Niger and Senegal from 2010-2012 in the second series of trials, resulting in five pairs of –P and + P environments. Set 3 genotypes, consisting of Sudanian zone adapated genotypes (56 researcher bred and 4 landrace varieties), was grown at a single Sudanian zone location in Mali in 2011 and 2012, providing two pairs of –P and + P environments. Four late maturing genotypes in set 1 were dropped from the analyses due to severe grain losses due to sorghum midge (*Stenodiplosis sorghicola*) infestation. A number of the trials conducted were not used in this analysis due to crop failure, heterogeneous growth due to edaphic constraints other than low P, such as soil crusting and variable depth of soil hard-pan and planting errors.

All experiments were laid out in an α-design with four replicates. Plots consisted of two three-meter rows with 75-80 cm distance between rows and 30-40 cm between hills within rows. Hills were thinned to two plants, resulting in a total of 6-9 plants m^−2^. A single border row planted with a common genotype of medium height and maturity separated each test plot to minimize neighbor effects. Soil samples were taken from most field experiments and analyzed for plant available soil P (Bray-1P) [54], pH-H_2_O and subset of samples were tested for aluminum saturation (% Al of CEC) (Additional file 11: Table S3). The –P fields had an average Bray-1P value of 6.3 mg P kg^−1^soil, whereas the + P fields averaged 16.5 mg P kg^−1^soil. The –P and + P sites had similar pH values, which averaged 5.6.

### Phenotypic statistical analysis

Each single environment was separately analyzed for grain yield using a mixed model with genotypes as fixed and replications and incomplete blocks nested within replications as random. When this model had repeatability values below 0.75, several spatial models were fitted (see [55]) and the optimum model was selected based on its AIC value. Predicted values and standard errors were computed for each genotype in each environment and were used in a combined weighted two-stage analysis [5] with genotypes and environments as random within one fertility level, yielding best linear unbiased predictors (BLUPs) for each genotype. Genotypic variances differed within individual environments (Additional file 1: Table S1) and correlations among the various environments likewise differed (Additional file 2). Several variance and co-variance models (unstructured, diagonal, factorial) were fitted to model specific variance co-variance structures but they did not converge (e.g. unstructured model) or did not improve the model fit based on AIC estimates (e.g. diagonal, factorial). Therefore instead of these models a compound symmetry model was used for our analyses. For a higher computational efficiency, the variance components were first estimated using the AI algorithm within Genstat 15 and then used as starting values running the same model but using the Fisher-scoring option to estimate broad-sense heritability based on [56]. Further, a combined analysis across P-treatments was conducted with P-treatment as fixed and genotypes and environments (location x year-combination) as random to estimate the genotype-by-P interaction. Similar as in the analysis within each fertility level, we fitted several variance co-variance models. Convergence problems and lack of superiority of model fit based on AIC, lead us to a compound symmetry model.

Grain yield ratios (-P/+P) were estimated, whereby grain yield BLUPs of the –P trials were divided by the grain yield BLUPs of the + P trials. Genotypes showing a higher grain yield ratio are expected to be specifically adapted to –P conditions.

For possible mega-environment delineation and GxE visualization a genotype-genotype-by-environment (GGE) analysis was conducted for grain yield across all –P and + P sites using only grain yield data from the 8 common genotypes [57]. All data analyses were done with the Genstat 15 software.

### Genomic data

Total genomic DNA was extracted from a single 20 day old plant of each line by using DNeasy Plant Mini Kit (QIAGEN). Extracted DNA was checked for quality and quantity by Nanodrop and gel analyses. GBS libraries were prepared and analyzed at the Institute for Genomic Diversity (IGD), according to [20], using the enzyme ApeKI for digestion and creating a library with 188 unique barcodes. GBS libraries were sequenced on the Illumina HiSeq2000. The GBS analysis pipeline Version 3.0.121, an extension to the Java program TASSEL [58], was used to call SNPs from the sequenced GBS library. The sequenced tags were aligned to the sorghum reference genome BTx623 v1.0 [59] and only tags with at least 10x coverage were retained. Ambiguous or heterozygous sites were set as missing SNPs and finally imputed with all other missing SNPs using NPUTE [60] for each chromosome separately. Imputation accuracy was on average above 96%. In total 308 623 SNPs were retrieved. After filtering for 5% minor allele frequency (MAF) using TASSEL, 220 934 SNPs were retained and used for further analysis. Gene specific markers for the *Alt*_*SB*_ locus were created by EMBRAPA and converted to the KASPar system at LGC-Genomics [22]. All genotypes were analyzed for these markers.

### Population genetics

Pairwise linkage disequilibrium (LD) was calculated for each chromosome separately in TASSEL using a sliding window size of 50 SNPs. Average LD decay across each chromosome was calculated and plotted in R. LD within a specific genomic region was calculated and visualized using LDheatmap in R [61]. Population structure was estimated using principal component analysis (PCA) as implemented in SNPrelate [62] and Admixture [63]. Kinship (K) among genotypes was calculated using the EMMA algorithm within GAPIT [64]. Allele frequencies of each site were calculated in TASSEL.

### Association mapping

The grain yield BLUPs estimated across –P, +P, combined across all –P and + P sites and the grain yield ratios (–P/+P) were used in a genome wide association study (GWAS). GWAS was carried out in R using the package GenABEL [21] running several models using the polygenic function correcting only for kinship or for kinship and population structure using either one, two, three, five or ten PCAs. The best model was chosen based on the lambda estimate of the quantile-quantile plots of the expected versus the observed p-values, thus having the lowest genome-wide inflation. For most traits a model with one to three PCAs fitted best. Multiple testing was corrected using a Bonferroni threshold of p < 0.05 (p-value/SNPs). Association mapping for the *Alt*_*SB*_ specific markers was performed in TASSEL using a compressed mixed linear model correcting for kinship and three PCAs [65]. Possible causal changes of associated SNPs were tested by the Variant effect Predictor of the www.gramene.org website using the sorghum genome v.1.0.

## Additional files

Additional file 1: Table S1. Single environment analysis results: the different genotype sets (GenoSet), number of genotypes tested within each set (#Geno), environmental zone in which the experiments were conducted (Zone), name of the environment as site-year combination (Site-Year), phosphorus treatment (P-level), grand mean for grain yield (Mean), genetic variance component (σ^2^G), genetic coefficient of variation (GCV), residual variance component (σ^2^_*error*_), standardized variance of a difference across all genotype pairs (stdzaVD) and repeatability estimate (w^2^). Additional file 2
**GGE-Biplot of sorghum grain yield of eight genotypes evaluated across 29 environments (15 –P and 14 + P environments).** The environments are coded as: first three letters indicate the location, followed by the year and the P-treatment (LP = –P, HP = +P). Additional file 3
**Boxplots of –P/+P grain yield ratios of the seven main racial groups existing among the 187 sorghum genotypes from West Africa.**
Additional file 4
**Genotype classification including information on: the different genotype sets (GenoSet), the eight common genotypes (ComGeno), the genotype ID (Taxa), the variety name (Variety), the racial classification based on morphological characters (Race), the country of origin (Country) and the alleles of some of the most important SNPs identified in this study.**
Additional file 5
**Average linkage disequilibrium (LD) decay of each chromosome based on r** ^**2**^ **estimates.** LD estimates were based on ~220 k SNPs derived from genotyping-by-sequencing and 187 West African sorghum genotypes. Additional file 6
**Population structure based on principal components 1 and 2 of the 187 sorghum genotypes with their corresponding morphological race classification.**
Additional file 7
**Hierarchical population structuring using Admixture assuming K = 3, 5 and 7.** K = 5 was best model based on error of cross-validations. The 187 sorghum genotypes are shown with their variety name and their corresponding morphological race classification. Additional file 8
**Fitted values (BLUPs) of the genotype groups carrying the most significant SNPs on Chr3 and Chr7 for grain yield across –P and + P conditions combined and on Chr1 and Chr5 for grain yield ratio.**
Additional file 9
**Manhattan plots of grain yield in –P (A), grain yield in + P (B) and grain yield ratio –P/+P (C).** *P* values are shown on a log_10_ scale. Additional file 10: Table S2. Results of most significant SNPs for grain yield in –P (GY–P), +P (GY + P), across both P-treatments (GY_ALL) and for grain yield ratios (–P/+P) (Ratio). Shown are: SNP ID, its position (Pos) on chromosome (Chr) of the sorghum reference genome v1.4 and the reference and alternative allele at this SNP (Allele), its p-value in GWAS, the explained genotypic variance (R^2^%), the consequence (CQs), the amino acid change (AA), the codon change (Codon), the distance in base pairs to another gene in close vicinity (<50 kb), the ID of this gene (Gene) and its predicted function based on different databases as summarized on www.phytozome.com. Additional file 11: Table S3. Single environment characteristics: sowing date, total amount of rain in whole year (mm), amount of N and P applied as fertilizer (kg/ha), pH of the soil measured in water (pH), soil content of plant available P measured as Bray-1P (mg P/kg soil), total amount of P in the soil (mg P/kg), aluminum saturation of the soil as percent of cat ion exchange capacity (CEC) (Al^3+^-sat.), timing of soil sampling (SS) and the amount and type of extra fertilizer added per hectar. Additional file 12
**Map of field trial locations with isohytes.** Locations circled and highlighted are Sahelian sites, while others are located in the Sudanian zone of West Africa.
