# Supplementary material for: Two in one sweep: aluminum tolerance and grain yield in P-limited soils are associated to the same genomic region in West African Sorghum
Source: BMC Plant Biol. 2014 Aug 12;14:206. doi: 10.1186/s12870-014-0206-6 (PMC4256928; doi:10.1186/s12870-014-0206-6)
Supplement: Additional file 1: Table S1. — Single environment analysis results: the different genotype sets (GenoSet), number of genotypes tested within each set (#Geno), environmental zone in which the experiments were conducted (Zone), name of the environment as site-year combination (Site-Year), phosphorus treatment (P-level), grand mean for grain yield (Mean), genetic variance component (σ2G), genetic coefficient of variation (GCV), residual variance component (σ2 error), standardized variance of a difference across all genotype pairs (stdzaVD) and repeatability estimate (w2). [file 12870_2014_206_MOESM1_ESM.pdf]

Table S1: Single environment analysis results: the different genotype sets (GenoSet), number of genotypes tested within each set (#Geno), environmental zone in which the experiments were conducted (Zone), name of the environment as site-year combination (Site-Year), phosphorus treatment (P-level), grand mean for grain yield (Mean), genetic variance component (σ^2G), genetic coefficient of variation (GCV), residual variance component (σ^2_{error}), standardized variance of a difference across all genotype pairs (stdzaVD) and repeatability estimate (w^2).

GenoSet	#Geno	Zone†	Site-Year	P-level	Mean	σ^2G	GCV	σ^2_{error}	stdzaVD	w^2‡
1	70	Sud	Kolombada2006	+P	115.80	489.00	19.10	1015.00	18.00	0.69
1	70	Sud	Kolombada2006	-P	84.60	267.80	19.30	756.60	18.40	0.69
1	70	Sud	Kolombada2007	+P	85.40	848.30	34.10	709.80	21.70	0.83
1	70	Sud	Kolombada2007*	-P	35.20	1.69	21.90	0.11	18.20	0.74
1	70	Sud	Kolombada2008	+P	157.30	578.20	15.30	770.40	11.70	0.77
1	70	Sud	Kolombada2009	+P	177.40	1953.00	24.90	2052.00	13.60	0.87
1	70	Sud	Kolombada2009	-P	100.50	1026.30	31.90	990.60	25.50	0.76
1	70	Sud	Samanko2006	+P	192.20	1852.00	22.40	1050.00	13.00	0.86
1	70	Sud	Samanko2006	-P	118.80	1370.00	31.20	1067.00	16.90	0.87
1	70	Sud	Samanko2007	-P	37.10	162.50	34.40	196.90	31.50	0.70
1	70	Sud	Samanko2008	-P	111.60	709.30	23.90	988.30	18.50	0.77
1	70	Sud	Samanko2009	+P	299.70	4910.00	23.40	4113.00	14.40	0.84
1	70	Sud	Samanko2009	-P	130.00	2475.00	38.30	1877.00	22.10	0.86
1	70	Sud	Samanko2010	+P	202.00	1817.00	21.10	1677.00	14.40	0.81
1	70	Sud	Samanko2010	-P	98.10	797.00	28.80	1099.00	22.00	0.77
2	80	Sah	Bema2010	+P	249.80	4689.00	27.41	2340.00	14.98	0.87
2	80	Sah	Bema2010	-P	141.10	3091.00	39.39	1899.00	23.09	0.85
2	80	Sah	Bambey2012	+P	95.22	2088.00	47.99	1069.00	25.09	0.88
2	80	Sah	Bambey2012	-P	61.69	893.70	48.46	579.20	29.82	0.84
2	80	Sah	Ssa-Maradi2012*	+P	10.00	0.20	45.02	0.15	28.37	0.83
2	80	Sah	Ssa-Maradi2012*	-P	4.05	0.15	64.59	0.17	53.15	0.75
2	80	Sah	Samanko2012	+P	137.00	1714.00	30.23	1315.00	19.66	0.83
2	80	Sah	Samanko2012	-P	109.10	818.00	26.22	544.50	15.92	0.84
2	80	Sah	Bema2012	+P	106.90	2137.00	43.26	2206.00	31.81	0.79
2	80	Sah	Bema2012	-P	87.17	1215.00	39.99	2509.00	40.91	0.66
3	60	Sud	Samanko2011	+P	258.30	3134.00	21.67	3381.00	17.97	0.74
3	60	Sud	Samanko2011	-P	111.90	1494.00	34.54	980.30	21.36	0.84
3	60	Sud	Samanko2012	+P	216.20	5285.00	33.63	4372.00	22.64	0.82
3	60	Sud	Samanko2012	-P	157.00	2085.00	29.09	1332.00	17.34	0.85

†Sud= Sudanian, Sah=Sahelian (see Figure S10); *data transformation applied, hence small variance components, means were back-transformed. ‡Equation 19 in Piepho, H.-P., and J. Möhring. 2007. Computing heritability and selection response from unbalanced plant breeding trials. Genetics. 177: 1881–1888. doi:10.1534/genetics.107.074229.
