# Supplementary figures and images for: Two in one sweep: aluminum tolerance and grain yield in P-limited soils are associated to the same genomic region in West African Sorghum
Source: BMC Plant Biol. 2014 Aug 12;14:206. doi: 10.1186/s12870-014-0206-6 (PMC4256928; doi:10.1186/s12870-014-0206-6)

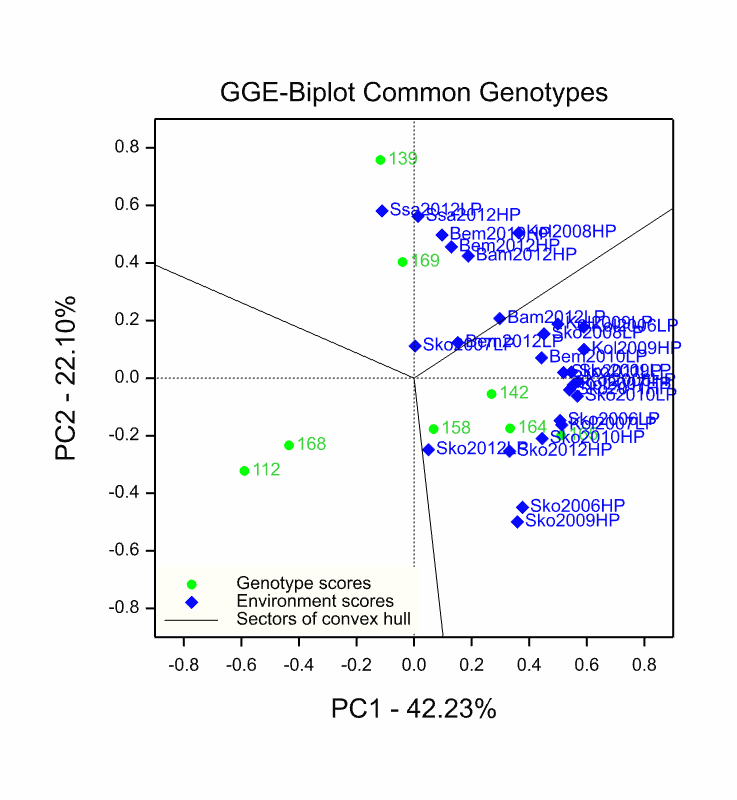

Supplement: Additional file 2 — GGE-Biplot of sorghum grain yield of eight genotypes evaluated across 29 environments (15 –P and 14 + P environments). The environments are coded as: first three letters indicate the location, followed by the year and the P-treatment (LP = –P, HP = +P). [file 12870_2014_206_MOESM2_ESM.png]

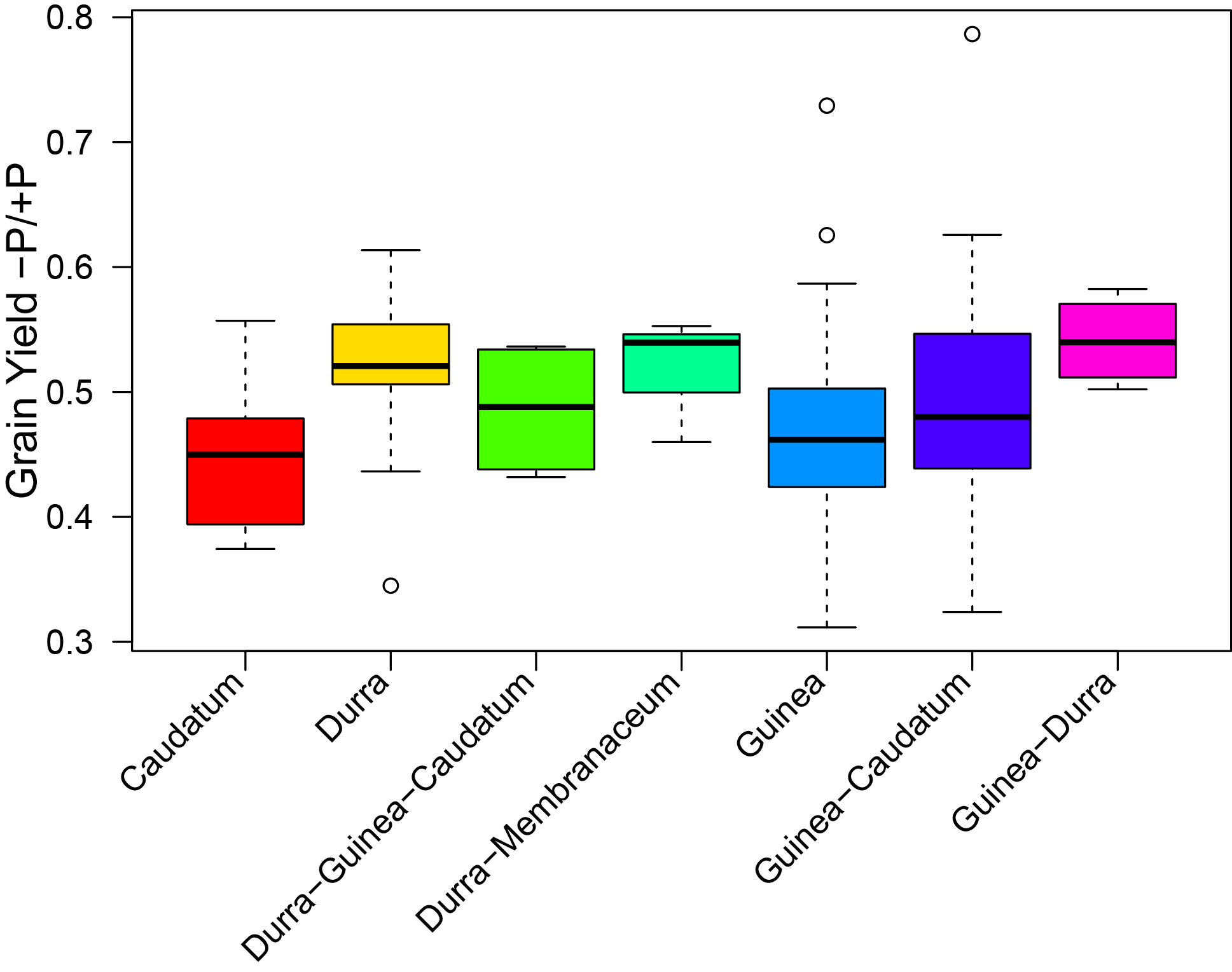

Supplement: Additional file 3 — Boxplots of –P/+P grain yield ratios of the seven main racial groups existing among the 187 sorghum genotypes from West Africa. [file 12870_2014_206_MOESM3_ESM.png]

Chr1

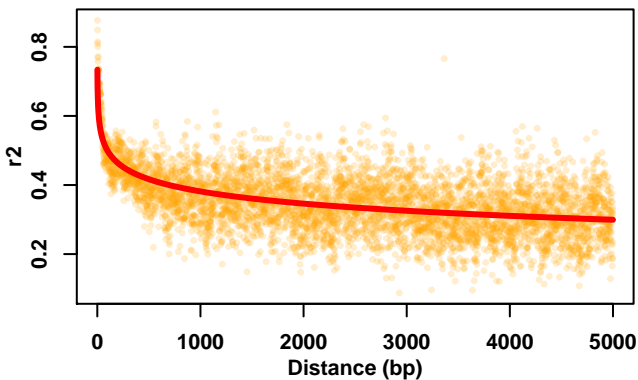

Chr2

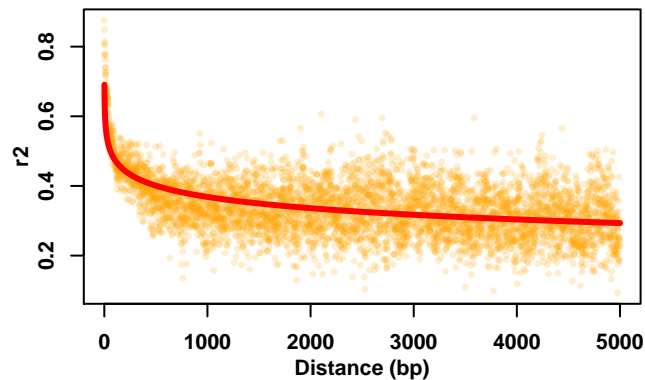

Chr3

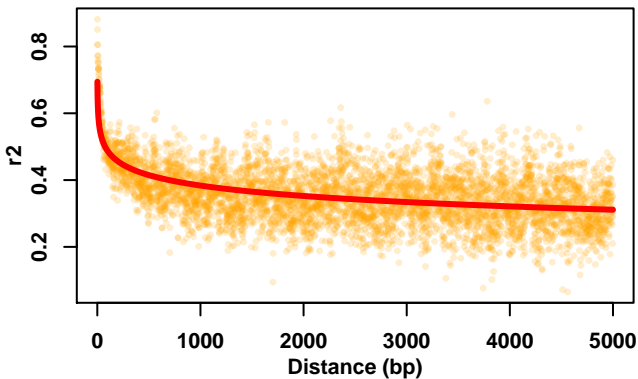

Chr4

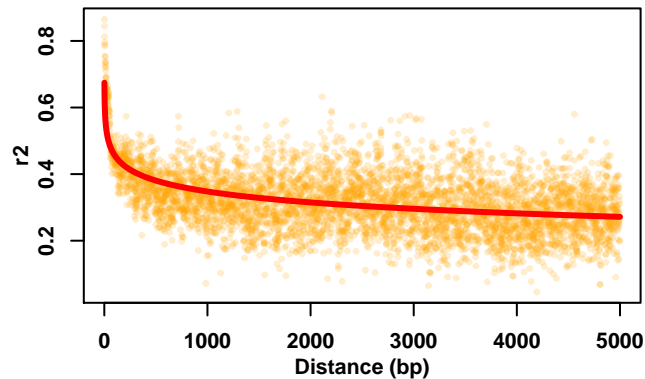

Chr5

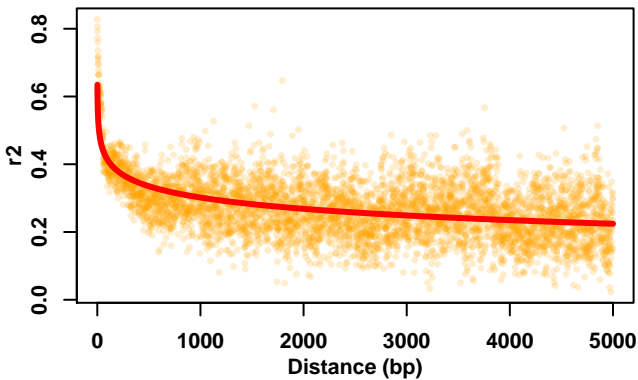

Chr6

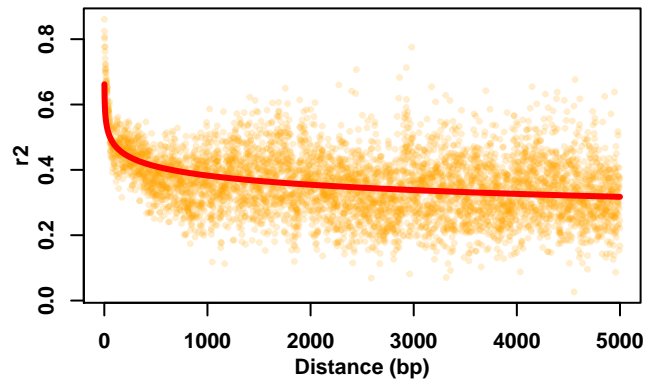

Chr7

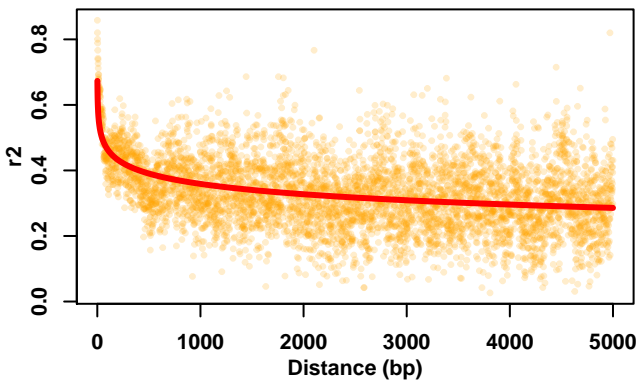

Chr8

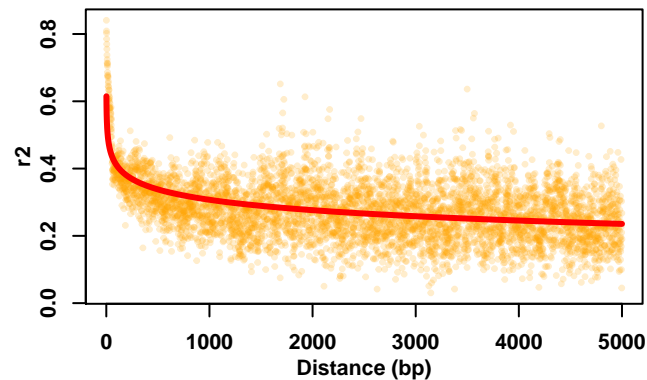

Chr9

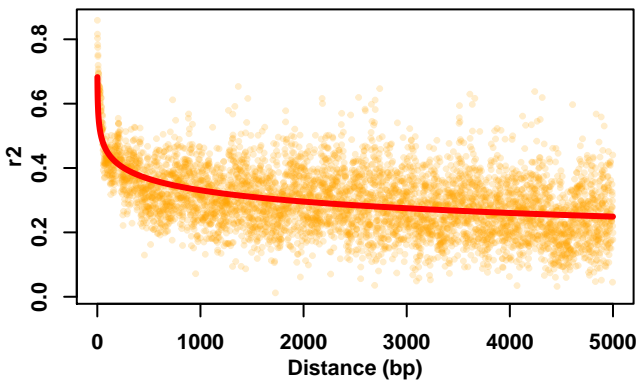

Chr10

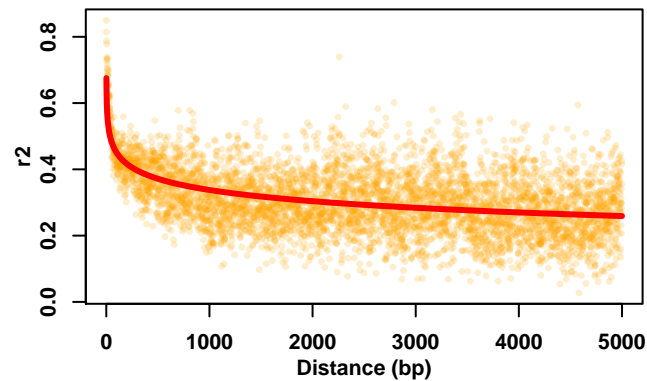

Supplement: Additional file 5 — Average linkage disequilibrium (LD) decay of each chromosome based on r 2 estimates. LD estimates were based on ~220 k SNPs derived from genotyping-by-sequencing and 187 West African sorghum genotypes. [file 12870_2014_206_MOESM5_ESM.pdf]

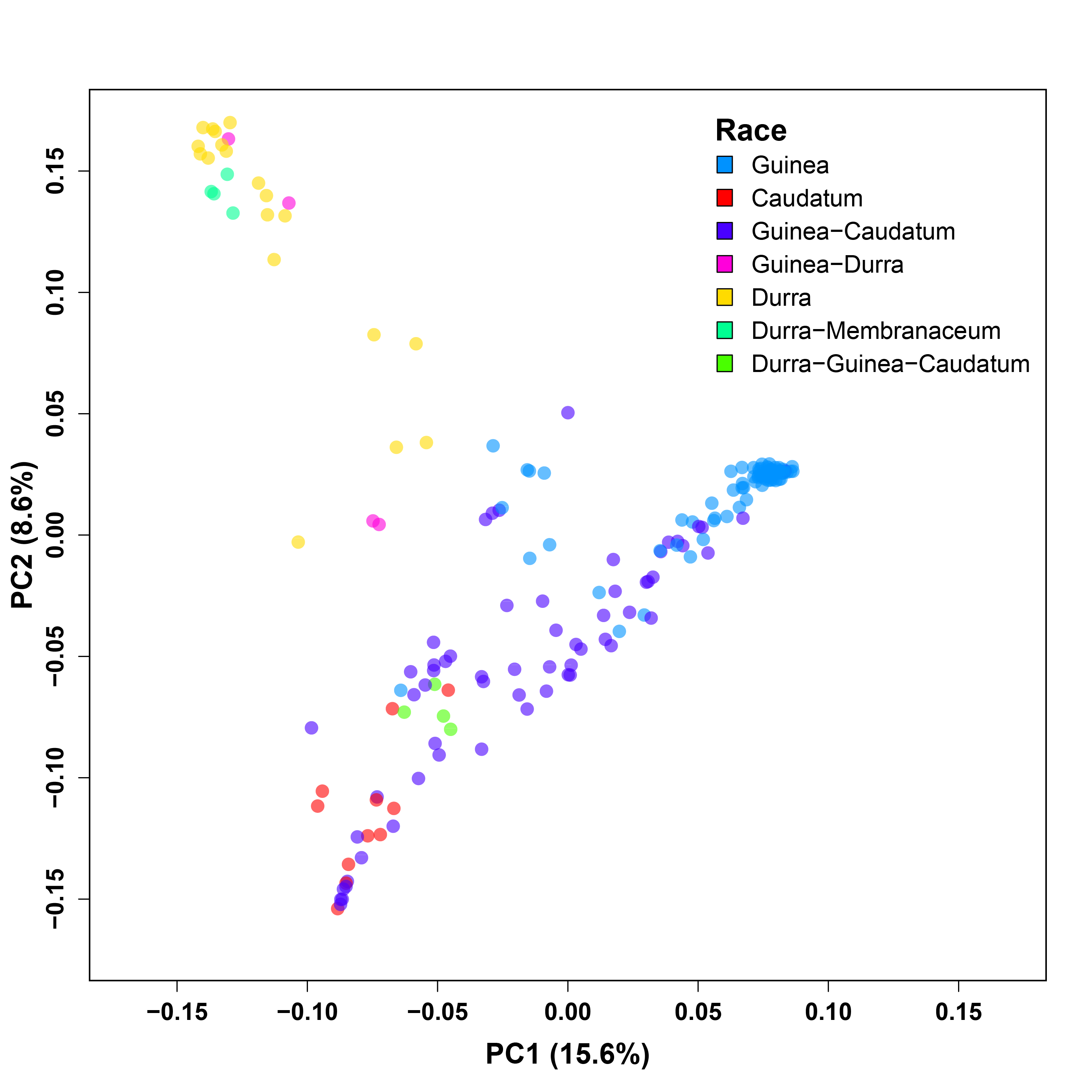

Supplement: Additional file 6 — Population structure based on principal components 1 and 2 of the 187 sorghum genotypes with their corresponding morphological race classification. [file 12870_2014_206_MOESM6_ESM.png]

K = 3

K = 5

K = 7

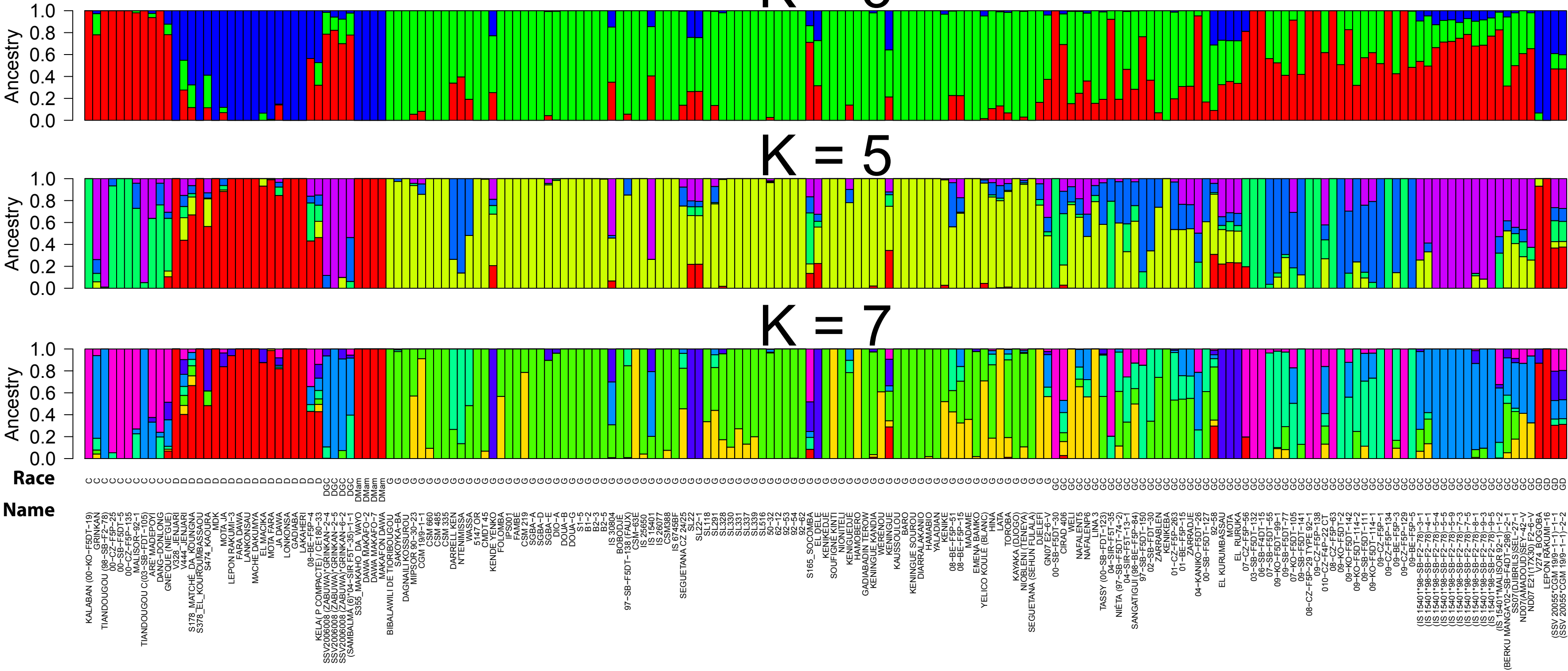

Supplement: Additional file 7 — Hierarchical population structuring using Admixture assuming K = 3, 5 and 7. K = 5 was best model based on error of cross-validations. The 187 sorghum genotypes are shown with their variety name and their corresponding morphological race classification. [file 12870_2014_206_MOESM7_ESM.pdf]

S3\_71101374

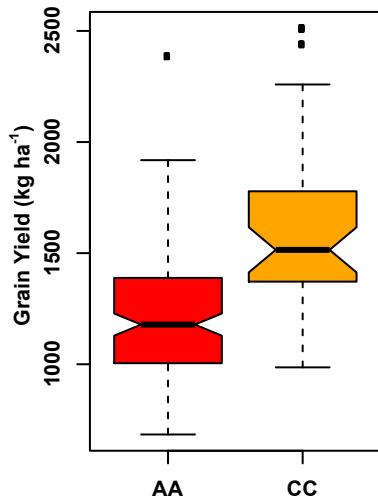

S3\_71103756

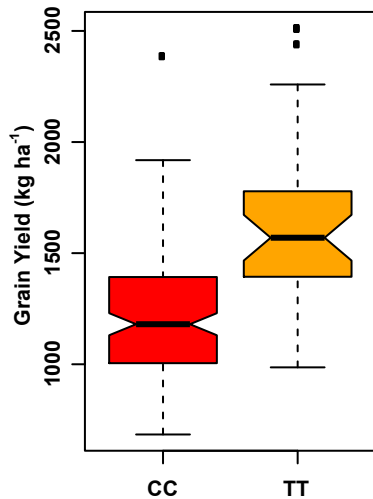

S3\_71178053

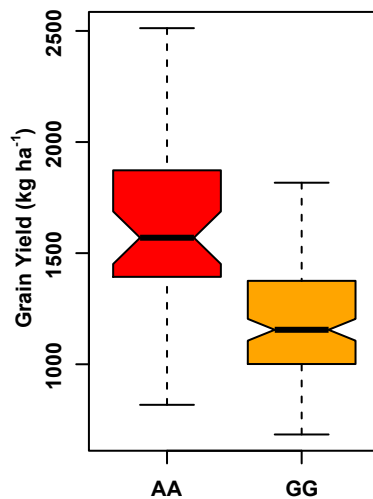

S7\_57969128

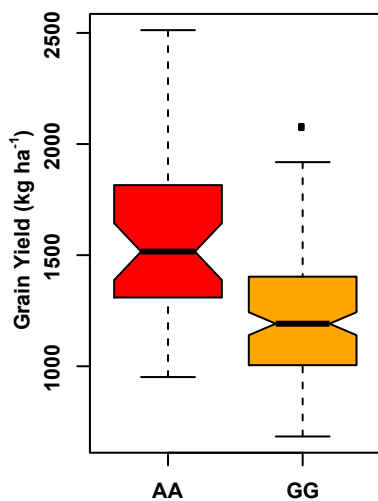

S1\_54947742

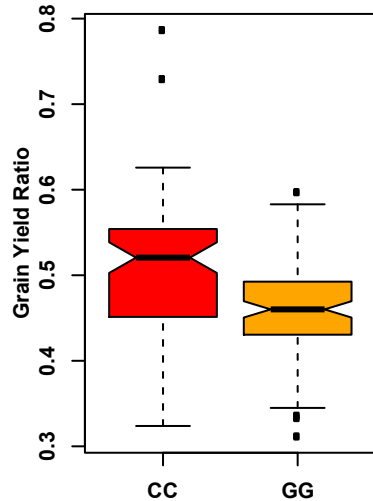

S5\_2179409

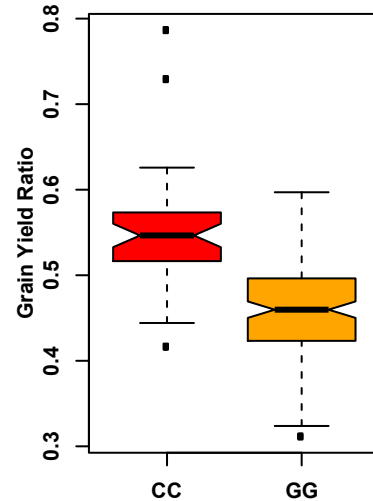

Supplement: Additional file 8 — Fitted values (BLUPs) of the genotype groups carrying the most significant SNPs on Chr3 and Chr7 for grain yield across –P and + P conditions combined and on Chr1 and Chr5 for grain yield ratio. [file 12870_2014_206_MOESM8_ESM.pdf]

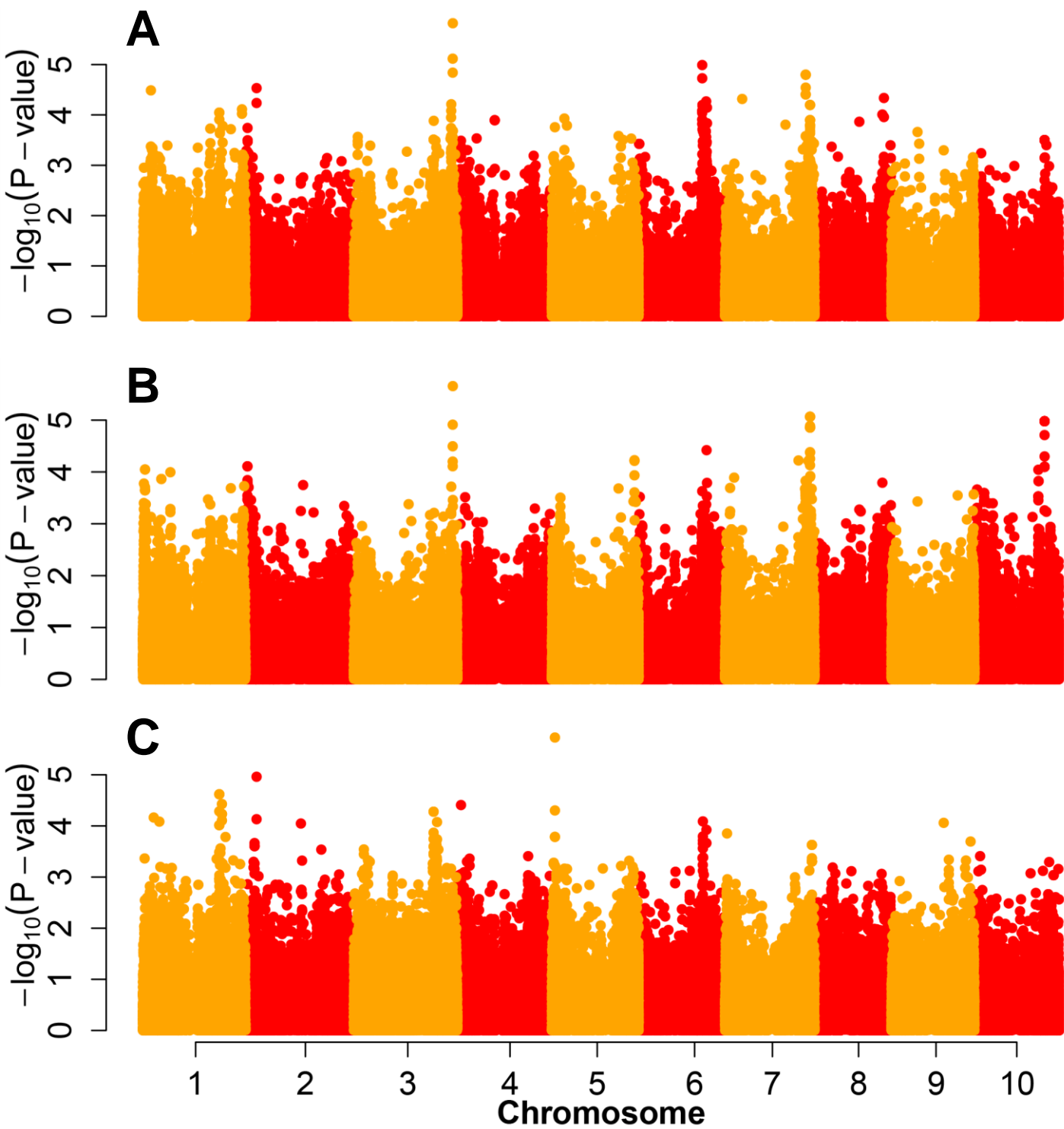

Supplement: Additional file 9 — Manhattan plots of grain yield in –P (A), grain yield in + P (B) and grain yield ratio –P/+P (C). P values are shown on a log10 scale. [file 12870_2014_206_MOESM9_ESM.pdf]

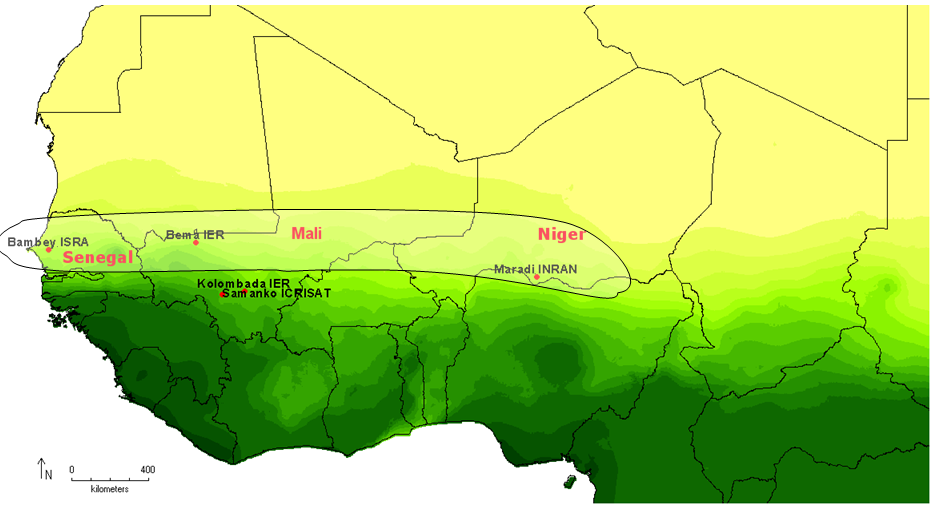

Supplement: Additional file 12 — Map of field trial locations with isohytes. Locations circled and highlighted are Sahelian sites, while others are located in the Sudanian zone of West Africa. [file 12870_2014_206_MOESM12_ESM.png]
