# Supplementary material for: Two in one sweep: aluminum tolerance and grain yield in P-limited soils are associated to the same genomic region in West African Sorghum
Source: BMC Plant Biol. 2014 Aug 12;14:206. doi: 10.1186/s12870-014-0206-6 (PMC4256928; doi:10.1186/s12870-014-0206-6)
Supplement: Additional file 10: Table S2. — Results of most significant SNPs for grain yield in –P (GY–P), +P (GY + P), across both P-treatments (GY_ALL) and for grain yield ratios (–P/+P) (Ratio). Shown are: SNP ID, its position (Pos) on chromosome (Chr) of the sorghum reference genome v1.4 and the reference and alternative allele at this SNP (Allele), its p-value in GWAS, the explained genotypic variance (R2%), the consequence (CQs), the amino acid change (AA), the codon change (Codon), the distance in base pairs to another gene in close vicinity (<50 kb), the ID of this gene (Gene) and its predicted function based on different databases as summarized on www.phytozome.com. [file 12870_2014_206_MOESM10_ESM.pdf]

Table S2: Results of most significant SNPs for grain yield in –P (GY–P), +P (GY+P), across both P-treatments (GY_ALL) and for grain yield ratios (–P/+P) (Ratio). Shown are: SNP ID, its position (Pos) on chromosome (Chr) of the sorghum reference genome v1.4 and the reference and alternative allele at this SNP (Allele), its p-value in GWAS, the explained genotypic variance (R^2 %), the consequence (CQs), the amino acid change (AA), the codon change (Codon), the distance in base pairs to another gene in close vicinity (<50kb), the ID of this gene (Gene) and its predicted function based on different databases as summarized on www.phytozome.com

**Trait**	**SNP ID**	**Chr**	**Pos**	**Allele**	**p-value**	**R^2 (%)**	**CQs***	**AA**	**Codon**	**Distance (bp)**	**Gene**	**Predicted Function**
Ratio	S1_54947742	1	54947742	G/C	2.69E-05	9.54	MSv	I/M	atC/atG		Sb01g032090	Os09g0381400 protein; Papain family cysteine protease
Ratio	S5_2179409	5	2179409	G/C	2.15E-06	12.15	INv				Sb05g001996	similar to LG27/30-like; Rhamnogalacturonate lyase family
Ratio	S2_7997952	2	7997952	T/G	1.24E-05	10.34	UsGv			<2000	Sb02g006380	similar to IN1
Ratio	S2_7997952	2	7997952	T/G	1.24E-05	10.34	DsGv			<300	Sb02g006370	similar to Kinase associated protein phosphatase
GY–P	S3_71178053	3	71178053	G/A	2.40E-06	12.37	MSv	A/T	Gcg/Acg		Sb03g043930	Putative uncharacterized protein
GY–P	S3_71101374	3	71101374	A/C	1.14E-05	10.71	DsGv			<400	Sb03g043840	Putative uncharacterized protein; P21-Rho-binding domain
GY–P	S3_71101374	3	71101374	A/C	1.14E-05	10.71	UsGv			<1500	Sb03g043850	similar to Zinc finger (C3HC4-type RING finger)-like
GY–P	S6_45739640	6	45739640	T/C	1.52E-05	10.41	INv				Sb06g016670	Ribonuclease p 25kda subunit-related
GY–P	S6_45739640	6	45739640	T/C	1.52E-05	10.41	UsGv			<4000	Sb06g016680	Putative uncharacterized protein
GY–P	S6_45739640	6	45739640	T/C	1.52E-05	10.41	DsGv			<8500	Sb06g016660	cop9 signalosome complex subunit 8
GY–P	S7_57976035	7	57976035	T/A	2.32E-05	9.96	INv				Sb07g023130	similar to NADPH HC toxin reductase
GY+P	S3_71178053	3	71178053	G/A	3.54E-06	11.98	MSv	A/T	Gcg/Acg		Sb03g043930	Putative uncharacterized protein
GY+P	S3_71178053	3	71178053	G/A	3.54E-06	11.98	DsGv			<6000	Sb03g043920	similar to Lysine ketoglutarate reductase trans-splicing related 1-like
GY+P	S3_71178053	3	71178053	G/A	3.54E-06	11.98	UsDv			<17000	Sb03g043940	Ubiquitin and ubiquitin-like proteins
GY+P	S7_61100380	7	61100380	G/A	1.31E-05	10.59	5'UTRv				Sb07g025960	Putative uncharacterized protein
GY+P	S7_61100380	7	61100380	G/A	1.31E-05	10.59	UsDv			<5000	Sb07g025970	similar to Pyruvate dehydrogenase E1 beta subunit isoform 3
GY+P	S7_61100380	7	61100380	G/A	1.31E-05	10.59	UsDv			<26000	Sb07g025980	similar to Putative diphosphonucleotide phosphatase
GY+P	S10_50433130	10	50433130	A/G	1.58E-05	10.38	DsGv			<3500	Sb10g022510	Putative uncharacterized protein
GY+P	S10_50433130	10	50433130	A/G	1.58E-05	10.38	UsGv			<15000	Sb10g022520	Cytochrome P450 CYP2 subfamily
GY_ALL	S3_71178053	3	71178053	G/A	1.75E-07	15.36					shown above	
GY_ALL	S3_71101374	3	71101374	A/C	3.07E-06	12.25					shown above	
GY_ALL	S7_57976035	7	57976035	T/A	9.38E-06	11.05					shown above	
GY_ALL	S7_61100380	7	61100380	G/A	1.19E-05	10.79					shown above	
GY_ALL	S6_45739640	6	45739640	T/C	2.07E-05	10.20					shown above	

* MSv=missense-variant, INv=intron-variant, UsGv=upstream-gene-variant, DsGv=downstream-gene-variant, 5'UTRv=5'-UTR-variant
