# Supplementary material for: Two in one sweep: aluminum tolerance and grain yield in P-limited soils are associated to the same genomic region in West African Sorghum
Source: BMC Plant Biol. 2014 Aug 12;14:206. doi: 10.1186/s12870-014-0206-6 (PMC4256928; doi:10.1186/s12870-014-0206-6)
Supplement: Additional file 11: Table S3. — Single environment characteristics: sowing date, total amount of rain in whole year (mm), amount of N and P applied as fertilizer (kg/ha), pH of the soil measured in water (pH), soil content of plant available P measured as Bray-1P (mg P/kg soil), total amount of P in the soil (mg P/kg), aluminum saturation of the soil as percent of cat ion exchange capacity (CEC) (Al3+-sat.), timing of soil sampling (SS) and the amount and type of extra fertilizer added per hectar. [file 12870_2014_206_MOESM11_ESM.pdf]

Table S3: Single environment characteristics: sowing date, total amount of rain in whole year (mm), amount of N and P applied as fertilizer (kg/ha), pH of the soil measured in water (pH), soil content of plant available P measured as Bray-1P (mg P/kg soil), total amount of P in the soil (mg P/kg), aluminum saturation of the soil as percent of cat ion exchange capacity (CEC) (Al^{3+}-sat.), timing of soil sampling (SS) and the amount and type of extra fertilizer added per hectar.

GenoSet	Site-Year	P-level	Sowing date	Rain Total	N-fert	P-fert	pH	Bray1-P	total P	Al^{3+}-sat.	SS*	extra
1	Kolombada2006	+P	07.18.06	906.0	41.0	20.0	n.a.	16.6	n.a.	n.a.	a.s.	
1	Kolombada2006	-P	07.18.06	906.0	23.0	0.0	n.a.	11.4	n.a.	n.a.	a.s.	
1	Kolombada2007	+P	07.05.07	880.0	41.0	20.0	5.0	19.5	n.a.	14.3	a.s.	
1	Kolombada2007	-P	07.05.07	880.0	23.0	0.0	4.9	13.9	n.a.	21.0	a.s.	
1	Kolombada2008	+P	07.10.08	616.0	41.0	20.0	n.a.	16.5	n.a.	n.a.	a.s.	
1	Kolombada2009	+P	07.09.09	720.0	29.5	20.0	5.1	16.0	108.7	1.6	a.s.	
1	Kolombada2009	-P	07.09.09	720.0	23.0	0.0	5.3	8.9	105.7	2.1	a.s.	
1	Samanko2006	+P	06.27.06	1008.0	29.0	17.0	5.0	14.4	106.2	8.8	a.s.	
1	Samanko2006	-P	06.28.06	1008.0	46.0	0.0	4.4	6.5	122.1	20.6	a.s.	
1	Samanko2007	-P	07.16.07	809.0	46.0	0.0	n.a.	3.9	n.a.	n.a.	a.s.	
1	Samanko2008	-P	07.07.08	1035.0	46.0	0.0	n.a.	8.0	n.a.	n.a.	a.s.	
1	Samanko2009	+P	07.08.09	1068.0	59.0	40.0	5.2	18.8	111.6	10.7	a.s.	
1	Samanko2009	-P	07.08.09	1068.0	58.4	0.0	4.8	7.4	111.7	22.8	a.s.	
1	Samanko2010	+P	06.26.10	1230.0	41.0	20.0	5.7	19.2	n.a.	8.0	a.s.	
1	Samanko2010	-P	06.26.10	1230.0	40.9	0.0	6.4	5.7	n.a.	2.0	a.s.	
2	Bambey2012	+P	07.07.12	583.6	52.5	20.0	7.0	5.2	n.a.	7.5	b.s.	
2	Bambey2012	-P	07.07.12	583.6	52.4	0.0	6.8	3.1	n.a.	27.5	b.s.	
2	Bema2010	+P	07.20.10	509.5	50.2	20.0	6.0	1.6	n.a.	0.0	b.s.	
2	Bema2010	-P	07.19.10	509.5	50.1	0.0	5.9	2.0	n.a.	0.2	b.s.	
2	Bema2012	+P	07.09.12	564.5	52.5	20.0	n.a.	n.a.	n.a.	n.a.		
2	Bema2012	-P	07.09.12	564.5	52.4	0.0	n.a.	n.a.	n.a.	n.a.		
2	Samanko2012	+P	06.18.12	1105.4	70.5	40.0	n.a.	28.5	n.a.	n.a.	a.s.	14T com
2	Samanko2012	-P	06.18.12	1105.4	52.4	0.0	n.a.	4.8	n.a.	n.a.	a.s.	14T com
2	Ssa-Maradi2012	+P	06.23.12	546.5	52.5	20.0	5.6	3.5	n.a.	n.a.	b.s.	
2	Ssa-Maradi2012	-P	06.23.12	546.5	46.0	0.0	5.6	3.5	n.a.	n.a.	b.s.	
3	Samanko2011	+P	06.25.11	1019.9	47.5	40.0	6.0	25.6	n.a.	9.6	a.s.	
3	Samanko2011	-P	06.25.11	1019.9	29.4	0.0	6.6	5.2	n.a.	1.7	a.s.	
3	Samanko2012	+P	06.18.12	1105.4	70.5	40.0	n.a.	28.5	n.a.	n.a.	a.s.	14T com
3	Samanko2012	-P	06.18.12	1105.4	52.4	0.0	n.a.	4.6	n.a.	n.a.	a.s.	14T com

*= soil sampling time; a.s.= after sowing, b.s.= before sowing. n.a. = missing values. 14T com= 14 tonnes/ha of compost applied before sowing.
